# Evaluation of Drug-Induced Liver Injury in Hospitalized Patients with SARS-CoV-2 Infection

**DOI:** 10.3390/microorganisms10102045

**Published:** 2022-10-17

**Authors:** Nicoleta Mihai, Catalin Tiliscan, Constanta Angelica Visan, Laurentiu Stratan, Oana Ganea, Stefan Sorin Arama, Mihai Lazar, Victoria Arama

**Affiliations:** 1 Faculty of Medicine, “Carol Davila” University of Medicine and Pharmacy, 37 Dionisie Lupu Street, 020021 Bucharest, Romania; 2 “Prof. Dr. Matei Bals” National Institute for Infectious Diseases, 1 Calistrat Grozovici Street, 021105 Bucharest, Romania

**Keywords:** COVID-19, SARS-CoV-2, liver injury, tocilizumab, anakinra, favipiravir, remdesivir

## Abstract

Elevated liver enzymes are frequently reported in SARS-CoV-2-infected patients. Several mechanisms of liver injury have been proposed, but no clear conclusions were drawn. We aimed to evaluate hepatocellular and cholestatic injury in relation to the administration of potentially hepatotoxic drugs included in the current COVID-19 therapeutic guidelines in a retrospective cohort of 396 hospitalized COVID-19 patients. The main findings of our study are: (1) Significant increase in aminotransferases level was observed during hospitalization, suggesting drug-related hepatotoxicity. (2) Tocilizumab was correlated with hepatocellular injury, including ALT values greater than five times the upper limit of normal. (3) Anakinra was correlated with ALT values greater than three times the upper limit of normal. (4) Younger patients receiving tocilizumab or anakinra had a higher risk of hepatocellular injury. (5) The combination of favipiravir with tocilizumab was associated with AST values greater than three times the upper limit of normal and with an increase in direct bilirubin. (6) The administration of at least three potentially hepatotoxic drugs was correlated with hepatocellular injury, including ALT values greater than five times the upper limit of normal, and with the increase in indirect bilirubin. (7) Remdesivir and favipiravir by themselves did not correlate with hepatocellular or cholestatic injury in our study cohort.

## 1. Introduction

The current SARS-CoV-2 pandemic still represents a challenge for the healthcare system. Since December 2019, considerable progress has been made in terms of describing the structure of SARS-CoV-2, the underlying pathophysiological mechanisms, the diagnostic techniques, as well as the strategies for prevention and treatment. However, there are still unanswered questions, particularly given that the virus can rapidly develop mutations.

Although COVID-19 patients typically develop lung injury, multiple systems and organs can be involved during infection. Elevated liver enzymes are frequently observed in medical practice in SARS-CoV-2 infected patients, although the incidence reported in studies varies between 3.75% and 76.3% [1,2]. Until now, several mechanisms of liver injury have been hypothesized (direct viral damage, drug-induced hepatotoxicity, systemic inflammation, exacerbation of underlying liver disease, hypoxia), but it has not been possible to pinpoint to what extent each of them is involved [3].

We must note that therapeutic guidelines for SARS-CoV-2 infection include drugs with hepatotoxic potential. Thus, it is necessary to establish what is the contribution of drug hepatotoxicity to the liver injury encountered in COVID-19 patients and what precautions are required when using these drugs. Treatment guidelines underwent several changes throughout the pandemic. The initial regimens, lopinavir/ritonavir, hydroxychloroquine, and azithromycin, did not prove effective in the treatment of SARS-CoV-2. Currently, therapeutic resources include drugs such as remdesivir (RDV), favipiravir (FVP), tocilizumab (TCZ), and anakinra (ANK), all with hepatotoxic potential [4].

Until now, there are several studies that have indicated a connection between COVID-19 treatment and the occurrence of liver injury. A meta-analysis by Kulkarni et al. published in 2020 which included 107 articles (20,874 patients) showed a pooled incidence of drug-induced liver injury (DILI) in COVID-19 of 25.4% [5]. However, in this meta-analysis, as in many other studies, DILI was defined as any increase in liver enzymes or total bilirubin occurring after the initiation of COVID-19 treatment, with no additional supporting evidence pointing to the drugs’ involvement.

Additionally, many of the studies and reviews carried out thus far predominantly refer to treatments such as hydroxychloroquine and lopinavir/ritonavir, which are no longer used today [6,7,8]. Moreover, some studies evaluated current treatments such as RDV or TCZ on patients who were also receiving drugs that are no longer part of the current standard of care [9,10]. As a result, it is difficult to determine which drugs are responsible for the occurrence of hepatotoxicity.

Our aim was to describe the characteristics of hepatocellular and cholestatic injury in a cohort of SARS-CoV-2-infected patients hospitalized in a tertiary care unit from Bucharest, Romania, and to evaluate liver injury in relation to the administration of potentially hepatotoxic drugs included in the current COVID-19 therapeutic guidelines (RDV, FVP, TCZ, and ANK).

## 2. Materials and Methods

### 2.1. Study Design and Participants

We performed a retrospective cohort study, including consecutive patients hospitalized in “C XI”department of the “Prof. Dr. Matei Bals” National Institute of Infectious Diseases, Bucharest, Romania, from December 2020 to August 2021. The only inclusion criteria were age above 18 and confirmation of SARS-CoV-2 infection by rapid antigenic test or real-time polymerase chain reaction (RT-PCR). Patients who could not sign the informed consent on admission were excluded. The study was approved by the ethics committee of “Prof. Dr. Matei Bals” National Institute for Infectious Diseases (C14730/2021).

### 2.2. Data Collection

For the included patients, a Microsoft Office Excel database was completed including demographic data (age, sex), body mass index (BMI), comorbidities, liver disease history, blood oxygen saturation level on admission, the severity of COVID-19, clinical outcome, treatment administered during hospitalization, laboratory data: dynamic values (on admission, on discharge and peak value–defined below) of alanine aminotransferase (ALT), aspartate aminotransferase (AST), gamma-glutamyl transferase (GGT), alkaline phosphatase (ALP), direct bilirubin (DBIL), indirect bilirubin (IBIL) and lactic dehydrogenase (LDH), serum albumin, serum lipase (determined with VITROS 5.1 FS, VITROS 4600, Siemens Dimension EXL Chemistry Analyzer, RAMP 200), C-reactive protein (CRP, determined with Siemens BN ProSpec System), serological markers for hepatitis B and C viruses (HBV and HCV, respectively, determined with VITROS 3602 Immunodiagnostic Systems).

### 2.3. Definitions

We used the National Institutes of Health (NIH) classification of COVID-19 severity [4]:

- Mild illness: signs and symptoms of COVID-19, but without pulmonary involvement
- Moderate illness: pulmonary involvement, but with blood oxygen saturation level (SpO_2_) greater than 93% on room air
- Severe illness: pulmonary involvement and at least one of: SpO_2_ < 94%, respiratory rate over 30 breaths/min, the ratio of arterial partial pressure of oxygen to fraction of inspired oxygen (PaO_2_/FiO_2_) below 300 mm Hg, infiltrates in over 50% of the lungs.

We defined peak X (e.g., peak ALT) as the highest value of variable X documented in a patient during hospitalization, after initiation of COVID-19 therapy.

### 2.4. Statistical Analysis

We analyzed the variables that express liver injury in relation to the administration of potentially hepatotoxic drugs included in the current COVID-19 therapeutic guidelines (RDV, FVP, TCZ, ANK, and combinations of these drugs). We performed all analyses with IBM^®^ SPSS^®^ Statistics, Version 23.0, New York, NY, USA (released 2015). For the quantitative variables, we presented the mean and standard deviation (SD) and for the nominal and ordinal variables the frequencies. For bivariate analysis of non-normally distributed variables, we used the Mann–Whitney test. For dichotomous variables, we used the Chi-square test, presenting the odds ratio (OR) and the 95% confidence interval (95% CI) for it. Subsequently, the variables that were significantly correlated were entered into multivariate analysis, with adjustment for patient sex, age, BMI, CRP, blood oxygen saturation level, the severity of COVID-19 and pre-existing liver disease. For continuous dependent variables, we used linear regression. For dichotomous dependent variables, we used logistic regression, presenting the exposure coefficient and the 95% CI for it. We considered the limit of statistical significance to be *p* < 0.05.

## 3. Results

### 3.1. Characteristics of the Study Participants

We enrolled 396 patients with a mean (±SD) age of 58.3 ± 14.8 years (range 21–92 years), including 148 women (37.7%) with a mean age of 61.1 ± 14.3 years and 248 men (62.63%) with a mean age of 56.6 ± 14.9 years. More than three-quarters of patients (*n* = 330, 83.3%) had associated comorbidities. The most common were cardiovascular diseases (55.6%) and obesity (37.4%). Multiple comorbidities were reported in 93 patients (23.5%). Twenty-one patients (5.3%) had known pre-existing liver disease. For another eight patients, positive HBsAg (for two patients) and positive anti-HCV antibodies (for six patients) were detected on admission. In addition, 14 patients (3.5%) had an alcohol use disorder. Most patients developed a severe form of COVID-19 (*n* = 276, 69.7%). Five patients (1.26%) needed intubation and mechanical ventilation, while four patients (1%) died. The main characteristics of the study participants are summarized in Table 1.

### 3.2. COVID-19 Therapy Administered during Hospitalization

Almost all patients (*n* = 391, 98.7%) received at least one potentially hepatotoxic drug during hospitalization. A significant proportion of patients received treatment with RDV and/or FVP (53% and 50.3%, respectively) and 42.4% of patients received at least three potentially hepatotoxic drugs. In about half (51%) of the patients, the antiviral therapy was followed by the administration of immunomodulatory therapy. The combination of favipiravir with anakinra (FVP/ANK) was most frequently used, in 15.9% of patients. The COVID-19 treatment administered during hospitalization is systematized in Table 2.

### 3.3. Characteristics of Liver Injury in the Study Participants

In 295 (74.5%) patients, there was evidence of liver cytolysis. For 184 (46.5%) patients, it was present since admission. The degree of liver damage varied from grade 1 (mild), for 71.9% of patients with cytolysis, to grade 4 (acute hepatitis). No case of liver injury required intervention. In addition, in 25.5% of patients, we noted that AST increased predominantly over ALT. When we examined the dynamics of liver enzymes, we found that in 60.7% of patients, there was a significant increase in aminotransferases levels during hospitalization. A significant decrease in aminotransferases level occurred for 10.5% of patients, while 25.1% of patients had a stable evolution. We defined a significant increase or decrease in the level of aminotransferases as the transition from one degree of cytolysis to another in an increasing or decreasing manner, respectively. At discharge, 66% of patients with hepatocytolysis still had elevated aminotransferase levels.

Hepatic cholestasis was found in 176/387 (45.5%) of the patients, and 138/378 (36.5%) had high cholestasis enzyme values since admission. In the majority of these patients (87.5%), an isolated increase in GGT was detected. DBIL was elevated in 83/373 (22.2%) of patients. The highest detected value was 1.8 mg/dL. Only 6 patients out of 373 (1.6%) had elevated IBIL.

Hepatic cytolysis was associated with cholestasis in 159/389 patients (40.9%). In addition, liver cytolysis was associated with elevated lipase in 90/368 patients (24.4%). Furthermore, elevated LDH values were detected in the majority of patients (83.6%). Low albumin values were recorded in 68/346 patients (19.6%).

In total, 312 patients (78.78%) had liver injury (cytolysis and/or cholestasis). In Table 3, we systematized the characteristics of liver injury in the study participants.

### 3.4. Evaluation of Hepatocellular Injury in Relation to Potentially Hepatotoxic Drugs Included in the Current SARS-CoV-2 Therapeutic Guidelines

In bivariate analysis, peak ALT was significantly correlated with the administration of FVP, TCZ and ANK, as well as with the therapeutic combinations of favipiravir with anakinra (FVP/ANK), favipiravir with tocilizumab (FVP/TCZ) and remdesivir with tocilizumab (RDV/TCZ). In addition, peak ALT was correlated with the administration of at least three potentially hepatotoxic drugs from the SARS-CoV-2 therapeutic guideline. After multivariate analysis, correlations between peak ALT and TCZ, FVP/TCZ and administration of at least three potentially hepatotoxic drugs remained statistically significant. Analyzing the median of peak ALT in patients who received only TCZ compared to those who received FVP/TCZ, no statistically significant difference was observed between the two groups (*p* = 0.076). In addition, in bivariate analysis, we observed that peak ALT values greater than three times the upper limit of normal (ULN) were associated with the administration of TCZ, ANK, FVP/TCZ, RDV/TCZ and with the administration of at least three potentially hepatotoxic drugs. In multivariate analysis, only the correlations with ANK and with the administration of at least three potentially hepatotoxic drugs were maintained. Moreover, peak ALT values greater than five times ULN was correlated in multivariate analysis with the administration of TCZ, FVP/TCZ, RDV/TCZ, as well as with the administration of at least three potentially hepatotoxic drugs. However, in these patients, we found no statistically significant difference between the group that received only TCZ compared to the group that received FVP/TCZ (*p* = 0.197) or RDV/TCZ (*p* = 0.439).

Regarding peak AST, in bivariate analysis, it was significantly correlated with the administration of TCZ, FVP/TCZ, RDV/TCZ, as well as with the administration of at least three potentially hepatotoxic drugs. In multivariate analysis, correlations between peak AST and TCZ, FVP/TCZ and administration of at least three potentially hepatotoxic drugs remained statistically significant. However, analyzing the median of peak AST in patients who received only TCZ compared to those who received FVP/TCZ, no statistically significant difference was observed between the two groups (*p* = 0.123). In addition, peak AST values greater than three times ULN were correlated in bivariate analysis with the administration of FVP/TCZ, a correlation that was also maintained in multivariate analysis.

We also analyzed peak LDH in relation to potentially hepatotoxic drugs used to treat COVID-19. In bivariate analysis, peak LDH was correlated with the administration of RDV/TCZ, a correlation that was no longer maintained after multivariate analysis.

In Table 4 and Table 5, we systematized the results of the statistical analysis of hepatocellular injury in relation to COVID-19 therapy.

### 3.5. Evaluation of Cholestatic Injury in Relation to Potentially Hepatotoxic Drugs Included in the Current SARS-CoV-2 Therapeutic Guidelines

We analyzed peak GGT and peak ALP in relation to potentially hepatotoxic drugs used in COVID-19 treatment (RDV, FVP, ANK, TCZ), but we found no statistically significant correlation. However, peak DBIL was correlated, in bivariate analysis, with the administration of TCZ (*p* = 0.009) and FVP/TCZ (*p* = 0.018). After multivariate analysis, only the association between peak DBIL and FVP/TCZ remained statistically significant (*p* = 0.034, R = 0.318). The linear regression model included peak CRP and patient sex, which were also directly correlated with peak DBIL (*p* = 0.001 and *p* = 0.039, respectively). Moreover, the presence of a DBIL above the upper limit of normal was associated with the administration of FVP/TCZ with an exposure coefficient of 2.95 (95%CI = 1.20–7.22). Peak IBIL was correlated, in bivariate analysis, with TCZ (*p* = 0.001), FVP/TCZ (*p* = 0.015), RDV/TCZ (*p* = 0.022) and RDV/ANK (*p* = 0.035), as well as with the administration of at least three potentially hepatotoxic drugs (*p* = 0.000). After multivariate analysis, only the association with the administration of at least three potentially hepatotoxic drugs was maintained (*p* = 0.005, R = 0.344). The linear regression model included blood oxygen saturation level and patient sex, which were also significantly associated with peak IBIL (*p* = 0.031 and *p* = 0.005, respectively). Of these, oxygen saturation level had a negative coefficient.

## 4. Discussion

Our study found that elevated liver enzymes are common in SARS-CoV-2-infected patients. Although liver damage may already exist at the time of admission, we observed a significant increase in liver enzymes level during hospitalization, suggesting drug-related hepatotoxicity.

In addition, we noted the predominant increase in the value of AST over ALT in 25.5% of patients. This pattern has been reported before in COVID-19 patients, in absence of another specific context. The presence of mitochondrial dysfunction, SARS-CoV-2-induced hepatic steatosis, as well as altered hepatic perfusion secondary to microthrombosis are some of the mechanisms proposed to explain the predominance of AST in COVID-19 patients. If this pattern is observed during hospitalization (as we found in 11.6% of patients), drug-induced liver injury can also be an explanation [11].

We also found statistically significant correlations between the administration of COVID-19 treatment and the occurrence of elevated liver enzymes in hospitalized patients. We discuss these results below in relation to the existing studies to date, creating a section for each drug.

### 4.1. Tocilizumab (TCZ)

Tocilizumab (TCZ) is a recombinant monoclonal antibody blocking the interleukin 6 (IL-6) receptor. TCZ is primarily used to treat severe cases of rheumatoid arthritis. Currently, TCZ treatment has also been approved for use in patients with oxygen-demanding SARS-CoV-2 infection who are clinically deteriorating despite initiation of systemic corticosteroids and who have significantly elevated inflammatory markers [4].

Data related to hepatic adverse reactions of TCZ come mainly from studies in patients with rheumatic diseases receiving long-term treatment. An FDA review related to the efficacy and safety of TCZ in rheumatoid arthritis, which included five randomized, double-blind, controlled trials (product registration studies), showed that approximately 50% of patients receiving TCZ present increases in ALT and AST of 1–3 times ULN. If we consider only the patients who received TCZ monotherapy, 36% showed increases in ALT and 22% increases in AST of 1–3 times ULN. Only 1.8% and 1.1% had ALT and AST elevations above three times ULN, respectively. An increase in total bilirubin was detected in 8% of the patients [12]. In patients with rheumatoid arthritis, the peak level of aminotransferases was reached at 2 weeks after the initiation of treatment and then began to decrease until the administration of the next dose (week 4). Eight weeks after the final dose, liver enzyme values were close to the normal range [13]. Cases of clinically apparent liver damage accompanied by jaundice [14] or liver failure [15] have been rarely reported.

Regarding administration in patients with COVID-19, mild or moderate elevations of aminotransferases have been reported in 8–51% of patients receiving TCZ [10,16,17], but the majority of patients also received other COVID-19 drugs considered standard of care at that time. A multicenter, open-label randomized clinical trial conducted by Salvarani et al. in 2021 on 126 COVID-19 patients showed an increase in ALT in 8% of patients who received TCZ compared to 3% in those who received only standard of care [10]. The value of cholestasis enzymes was not generally reported in these studies, probably because TCZ is not known to have an important effect on them. In our study, TCZ was significantly correlated with hepatocellular injury (peak ALT and peak AST), including ALT values greater than five times the upper limit of normal. We did not find any correlation between the administration of TCZ and cholestatic injury, which is consistent with the existing data. Younger patients receiving TCZ had a higher risk of hepatocellular injury. Although we have not found a physiopathological explanation for this, a similar observation was reported in patients treated with antituberculous drugs [18]. Moreover, several studies reported that younger patients frequently have more hepatocellular damage. In contrast, it seems that older persons have a higher risk for cholestatic injury [19,20]. Various host factors, some age-related, can influence the risk of drug-induced liver injury: mitochondrial function, inflammation and immune responses, genetic polymorphisms associated with hepatotoxicity. In addition, physicochemical drug properties can also be related to age-specific liver injury [21]. However, the results of the studies carried out thus far do not seem to outline a clear physiopathological hypothesis related to drug-induced liver injury in young people.

The mechanism of liver injury in patients receiving TCZ is not known. It is assumed that this damage occurs due to the blocking of IL-6, which has an important role in liver regeneration. Another less likely hypothesis is that TCZ binds to IL-6 receptors in hepatocytes and activates complement-mediated or antibody-dependent cytotoxicity [12].

### 4.2. Anakinra (ANK)

Anakinra (ANK) is a recombinant interleukin 1 (IL-1) receptor antagonist that has an anti-inflammatory and immunomodulatory effect. ANK is mainly used in the treatment of rheumatoid arthritis and other inflammatory arthritides, but it has also been approved in the European Union for use in COVID-19 patients who require oxygen therapy [22].

Similar to TCZ, most of the information related to the safety of ANK comes from patients with rheumatic diseases. A multicenter, placebo-controlled trial conducted by Fleischmann et al. in 2003 on 1414 patients with rheumatoid arthritis mentions that no evidence of hepatotoxicity was identified in patients who received ANK for 24 weeks [23]. However, ANK has been associated with several cases of acute liver injury with a pattern of hepatocellular damage [24,25,26]. However, the reported cases are generally in patients with Still’s disease, in which liver injury may represent a manifestation of the disease itself [27]. Overall, the problem of hepatotoxicity has not attracted much attention, and many studies related to the safety of ANK do not mention any information about liver enzymes [28,29].

In patients with COVID-19, studies to date are also limited, probably because ANK is not yet widely used in the treatment of COVID-19. In a meta-analysis conducted in 2021, Somagutta et al. analyzed two cohort studies and one randomized controlled trial (in total, 143 patients received ANK and 115 standard of care). The authors concluded that the frequency of elevated liver enzymes was similar in the two groups [30]. In addition, a double-blind, randomized controlled trial conducted by Kyriazopoulou et al. later in 2021 on 594 COVID-19 patients (412 receiving standard of care plus ANK and 194 standard of care plus placebo) shows that the frequency of altered liver function tests following the administration of ANK is similar to that in the placebo group [31]. In contrast to these studies, we found that treatment with ANK was correlated with ALT values greater than three times the upper limit of normal. As with TCZ, younger patients had a higher risk of hepatocellular injury. In addition, we found no correlation between the administration of ANK and cholestatic injury.

The mechanism of liver injury caused by ANK is unknown. A possible starting point for future research could be related to the IL-1 receptor antagonist (IL-1RN) and IL-1β polymorphism, which has already been associated with antiretroviral hepatotoxicity [32,33].

### 4.3. Favipiravir (FVP)

Favipiravir (FVP) is a pyrazinecarboxamide derivative with an antiviral effect exerted by inhibiting viral RNA-dependent RNA polymerase. FVP was approved in Japan in 2014 for the treatment of influenza cases that were unresponsive to standard treatment [34]. Currently, FVP is used off label in the treatment of mild and moderate forms of COVID-19 [35].

Regarding the hepatic adverse reactions of FVP, a review carried out in 2020 by Pilkington et al. who analyzed six studies with a comparison group (with a total of 4299 participants), showed a frequency of elevated liver function tests of 2.1% in patients who received FVP compared to 2.4% in the comparison arm (patients who received another antiviral or placebo) [36]. In addition, a prospective study conducted by Doi et al. later in 2020, which included 82 patients who received FVP, showed a frequency of elevated ALT of 8.5%. These patients did not receive any other antiviral than FVP [37]. Not least, in a randomized clinical trial conducted in 2021 by Udwandia et al. on 150 patients, 6.8% of patients who received FVP plus supportive care showed increased liver enzyme values compared to 2.7% of patients who received only supportive care (antibiotics, antipyretics, vitamins) [38].

The mechanism of liver damage caused by FVP is not yet fully elucidated. In general, it is considered to be an idiosyncratic reaction [39]. However, a study conducted by Kawasuji et al. in 2021 on nine patients showed an association between the serum concentration of FVP and the occurrence of hepatotoxicity [40]. This suggests that the occurrence of FVP-induced liver injury may be dose-dependent. The patient reported by Yamazaki et al. with suspected cholestatic liver injury induced by FVP received a high dose of this drug: 6000 mg on the first day and then 2400 mg per day for a total of 14 days [41]. It is also speculated that the long duration of treatment could inhibit the metabolism of FVP into inactive metabolites, which would increase the risk of hepatotoxicity [39].

In our study, FVP treatment by itself did not correlate with liver damage. The lack of association could be related to the fact that the patients included in this study received a low dose of FVP (1600 mg on the first day and then 600 mg per day) for a brief length of time (6 days on average). However, we found that the combination of FVP with TCZ was associated with AST values greater than three times the upper limit of normal and with an increase in DBIL. Male patients with elevated CRP levels who received FVP/TCZ had a higher risk for increased DBIL.

### 4.4. Remdesivir (RDV)

Remdesivir (RDV) is a nucleoside analog that has an antiviral effect by inhibiting viral RNA-dependent RNA polymerase. RDV has been approved for the treatment of hospitalized COVID-19 patients as well as for the treatment of nonhospitalized patients with mild to moderate COVID-19 with risk factors for severe progression [4].

For the first time, an increase in aminotransferases was reported in phase I trials, in which RDV was administered to healthy volunteers. A higher frequency of ALT elevation was observed in subjects who received a higher dose (150 mg/day) and/or for a longer time (10–14 days). The aminotransferase elevations were mild to moderate, reversible, and asymptomatic. No increases in bilirubin and alkaline phosphatase were reported [42]. In observational studies that included patients with severe COVID-19, grade 3 and 4 elevations of transaminases were reported in up to 42.8% of patients who received RDV [43].

In randomized trials, the increase in transaminases in patients receiving RDV was similar to that in the comparative group [9,44,45]. The largest of these studies, conducted by Beijel et al. in 2020 on 1062 patients showed an increase in aminotransferases in 6% of patients receiving RDV compared to 10.7% in the placebo group [44]. In addition, Spinner et al. showed a frequency of ALT elevation in 34% of patients who received 5 days of RDV compared to 32% in those who received 10 days of RDV and 39% in those who received standard of care [45]. However, in all these studies, patients in all comparative arms also received standard of care (e.g., lopinavir/ritonavir, hydroxychloroquine), drugs that have a known hepatotoxic effect. This makes it difficult to appreciate to what extent the treatment with RDV caused the mentioned increase in liver enzymes. In our study, we found no correlation between RDV and hepatocellular or cholestatic injury.

Direct inhibition of mitochondrial RNA polymerase is the main mechanism incriminated in the liver damage caused by RDV. Another unconfirmed hypothesis is that of the occurrence of an idiosyncratic reaction [46].

### 4.5. Administration of at Least Three Potentially Hepatotoxic Drugs

It is already known that the administration of more than one potentially hepatotoxic drug greatly increases the risk of drug-induced liver injury [47]. In our study, we found that administration of at least three potentially hepatotoxic drugs was correlated with hepatocellular injury, with these patients having a 4.36 times higher risk of developing ALT values greater than 5 times ULN. In addition, male patients with low oxygen saturation levels on admission who received at least three potentially hepatotoxic drugs had a higher risk for increased IBIL.

Summarizing, the main findings of our study are: (1) Liver injury is a common extrapulmonary manifestation of hospitalized patients with SARS-CoV-2 infection. (2) Significant increase in aminotransferases level was observed during hospitalization, suggesting drug-related hepatotoxicity. (3) TCZ was significantly correlated with hepatocellular injury, including ALT values greater than five times the upper limit of normal. (4) ANK was correlated with ALT values greater than three times the upper limit of normal. (5) Younger patients receiving TCZ or ANK had a higher risk of hepatocellular injury. (6) We found no correlation between TCZ or ANK and cholestatic injury. (7) The combination of FVP with TCZ was associated with AST values greater than three times the upper limit of normal and with an increase in DBIL. (8) The administration of at least three potentially hepatotoxic drugs was correlated with hepatocellular injury, including ALT values greater than five times the upper limit of normal, and with an increase in IBIL. (9) RDV and FVP by themselves did not correlate with hepatocellular or cholestatic injury in our study cohort.

The strengths of our study include the fact that it is one of the few studies that evaluated liver injury in patients receiving only currently used COVID-19 treatment, results not being influenced by drugs that are no longer used. In addition, we took into account not only the isolated effect of each drug but also the impact of the administration of combinations of drugs. Thus, the study provides relevant updated information about drug-induced liver injury in real life. Another strength is represented by the performance of multivariate statistical analysis, with the adjustment of the results to patient sex, age, BMI, CRP, blood oxygen saturation level, the severity of COVID-19, and pre-existing liver disease.

Our study has some limitations. First, it has a retrospective design, with all the limitations given by this, and provides results from a single tertiary hospital. In addition, we did not take into account other potentially hepatotoxic drugs taken by patients for other conditions. Another limitation of the study is the relatively small number of patients.

## 5. Conclusions

Liver injury is a common extrapulmonary manifestation of hospitalized patients with SARS-CoV-2 infection. In this study, we showed a correlation between liver injury and some of the drugs included in the current COVID-19 therapeutic guidelines. We found that younger patients have a higher risk of drug toxicity. We recommend monitoring liver enzymes for all patients during hospitalization, in order to adjust the drug doses or reconsider the administration decision, if necessary. More studies are needed, especially large randomized controlled trials or prospective studies that provide evidence of causality based on the updated RUCAM score, for a better assessment of the risk of hepatotoxicity of COVID-19 drugs.

## Figures and Tables

**Table 1:** Characteristics of the study participants.

Variables	All Patients (N = 396)
Age (years), mean ± SD	58.3 ± 14.8
Male, *n* (%)	248 (62.6)
BMI (kg/m^2^), mean ± SD	28.4 ± 5.4
Comorbidity, *n* (%) Cardiovascular diseases Obesity Diabetes mellitus Chronic pulmonary diseases Pre-existing liver disease Chronic kidney diseases Active cancer Multiple comorbidities No comorbidities	330 (83.3) 220 (55.6) 148 (37.4) 72 (18.2) 34 (8.6) 29 (7.3) 23 (5.8) 15 (3.8) 93 (23.5) 66 (16.7)
Pre-existing liver disease, *n* (%) Chronic HBV infection Chronic HCV infection Unspecified liver tumor Polycystic liver disease Autoimmune liver disease Malignant bile duct obstruction HBV infection diagnosed on admission HCV infection diagnosed on admission	29 (7.3) 9 (2.3) 6 (1.5) 3 (0.75) 1 (0.25) 1 (0.25) 1 (0.25) 2 (0.5) 6 (1.5)
COVID-19 severity, *n* (%) Mild Moderate Severe	14 (3.5) 106 (26.8) 276 (69.7)
Outcome Discharge, *n* (%) Duration of hospitalization (days), mean ± SD IMV, *n* (%) Death, *n* (%)	391 (99) 10.7 ± 5.4 5 (1.26) 4 (1)

BMI, body mass index; HBV, hepatitis B virus; HCV, hepatitis C virus; IMV, invasive mechanical ventilation; SD, standard deviation.

**Table 2:** COVID-19 therapy administered during hospitalization.

COVID-19 Therapy	All Patients (N = 396)
LMWH/NOAC, *n* (%) RDV, *n* (%) Duration (days), mean ± SD FPV, *n* (%) Duration (days), mean ± SD ANK, *n* (%) Duration (days), mean ± SD Cumulative dose (mg), mean ± SD TCZ, *n* (%) No. of administrations, mean ± SD Cumulative dose (mg), mean ± SD FVP/ANK, *n* (%) RDV/TCZ, *n* (%) RDV/ANK, *n* (%) FVP/TCZ, *n* (%) At least 3 potentially hepatotoxic drugs, *n* (%)	390 (98.7) 210 (53) 4.76 ± 1.11 199 (50.3) 5.75 ± 2.61 102 (25.8) 5.25 ± 2.39 865.6 ± 408.2 81 (20.5) 1.98 ± 0.72 1066.33 ± 535.35 63 (15.9) 54 (13.6) 49 (12.4) 36 (9.1) 168 (42.4)

ANK, anakinra; FVP, favipiravir; FVP/ANK, combination therapy of favipiravir with anakinra; FVP/TCZ, combination therapy of favipiravir with tocilizumab; LMWH, low molecular weight heparin; NOAC, non-vitamin K oral anticoagulants; RDV, remdesivir; RDV/ANK, combination therapy of remdesivir with anakinra; RDV/TCZ, combination therapy of remdesivir with tocilizumab; TCZ, tocilizumab.

**Table 3:** Characteristics of liver injury in the study participants.

Variables	Total	On Admission	During Hospitalization
Liver injury (elevated hepatocytolysis and/or cholestasis enzymes), *n* (%)	312 (78.78)	225 (56.82)	277 (69.95)
Elevated aminotransferases (ALT and/or AST), *n* (%) Grade 1: (1–3) ×ULN Grade 2: [3–5) ×ULN Grade 3: [5–10) ×ULN Grade 4: ≥10 ×ULN	295 (74.5) 212 (53.54) 54 (13.64) 23 (5.81) 6 (1.51)	184 (46.47) 162 (40.91) 18 (4.55) 3 (0.76) 1 (0.25)	262 (66.16) 191 (48.23) 42 (10.6) 23 (5.81) 6 (1.52)
Elevated ALT, *n* (%) Grade 1: (1–3) ×ULN Grade 2: [3–5) ×ULN Grade 3: [5–10) ×ULN Grade 4: ≥10 ×ULN	275 (69.44) 198 (50) 49 (12.38) 22 (5.55) 6 (1.51)	142 (35.86) 126 (31.82) 14 (3.54) 1 (0.25) 1 (0.25)	257 (64.89) 189 (47.73) 40 (10.1) 22 (5.55) 6 (1.51)
Elevated AST, *n* (%) Grade 1: (1–3) ×ULN Grade 2: [3–5) ×ULN Grade 3: [5–10) ×ULN Grade 4: ≥10 ×ULN	204/395 (51.64) 180/395 (45.57) 19/395 (4.81) 5/395 (1.26) 0	147/387 (37.98) 135/387 (34.88) 9/387 (2.33) 3/387 (0.77) 0	144/364 (39.56) 128/364 (35.17) 13/364 (3.57) 3/364 (0.82) 0
(AST/ULN) > (ALT/ULN), *n* (%)	101 (25.5)	83 (20.96)	46 (11.62)
Elevated cholestasis enzymes, *n* (%) Isolated GGT GGT and ALP Isolated ALP	176/389 (45.24) 154/389 (39.59) 20/389 (5.14) 2/389 (0.51)	138/378 (36.51) 117/378 (30.95) 19/378 (5.03) 2/378 (0.53)	131/274 (47.81) 120/274 (43.8) 10/274 (3.65) 1/274 (0.36)
Elevated hepatocytolysis and cholestasis enzymes, *n* (%)	159/389 (40.87)	97/378 (25.66)	121/274 (44.16)
Elevated bilirubin, *n* (%) DBIL IBIL	83/373 (22.2) 6/373 (1.6)	62/373 (16.62) 4/361 (1.11)	35/216 (16.2) 4/215 (1.86)
Elevated LDH, *n* (%) Grade 1: (1–3) ×ULN Grade 2: [3–5) ×ULN Grade 3: ≥5 ×ULN	331 (83.59) 313 (79.04) 15 (3.79) 3 (0.76)	295 (74.5) 281 (70.96) 11 (2.78) 3 (0.76)	255/360 (70.83) 247/360 (68.61) 8/360 (2.22) 0

ALP, alkaline phosphatase; ALT, alanine aminotransferase; AST, aspartate aminotransferase; DBIL, direct bilirubin; GGT, gamma-glutamyl transferase; IBIL, indirect bilirubin; LDH, lactic dehydrogenase; ULN, upper limit of normal.

**Table 4:** Correlations between hepatocellular injury and COVID-19 therapy.

	Peak ALT		Peak AST		Peak LDH	
	Univariate Analysis	Multivariate Analysis	Univariate Analysis	Multivariate Analysis	Univariate Analysis	Multivariate Analysis
RDV	*p* = 0.446	-	*p* = 0.066	-	*p* = 0.595	-
FVP	*p* = 0.013 *	*p* = 0.426	*p* = 0.965	-	*p* = 0.667	-
ANK	*p* = 0.020 *	*p* = 0.053	*p* = 0.344	-	*p* = 0.829	-
TCZ	*p* = 0.001 *	*p* = 0.000 * R = 0.271 ^a^	*p* = 0.012 *	*p* = 0.033 * R = 0.112	*p* = 0.082	-
RDV/TCZ	*p* = 0.046 *	*p* = 0.137	*p* = 0.041 *	*p* = 0.115	*p* = 0.026 *	*p* = 0.404
RDV/ANK	*p* = 0.339	-	*p* = 0.337	-	*p* = 0.439	-
FPV/ANK	*p* = 0.011 *	*p* = 0.261	*p* = 0.157	-	*p* = 0.569	-
FVP/TCZ	*p* = 0.000 *	*p* = 0.003 * R = 0.285 ^b^	*p* = 0.006 *	*p* = 0.013 * R = 0.131	*p* = 0.218	-
3MED	*p* = 0.000 *	*p* = 0.000 * R = 0.312 ^b^	*p* = 0.000 *	*p* = 0.002 * R = 0.166	*p* = 0.648	-

* Statistically significant (*p* < 0.05); **^a^** The linear regression model included age (negative coefficient); **^b^** The linear regression model included age (negative coefficient) and CRP (positive coefficient); 3MED, at least three potentially hepatotoxic drugs; R, correlation coefficient.

**Table 5:** Correlations between grade 2 and grade 3 increase in liver cytolysis enzymes and COVID-19 therapy.

	Peak ALT ≥ 3×ULN		Peak ALT ≥ 5×ULN		Peak AST ≥ 3×ULN	
	Univariate Analysis	Multivariate Analysis	Univariate Analysis	Multivariate Analysis	Univariate Analysis	Multivariate Analysis
RDV	*p* = 0.275	-	*p* = 0.919	-	*p* = 0.946	-
FVP	*p* = 0.295	-	*p* = 0.245	-	*p* = 0.776	-
ANK	*p* = 0.017 * OR = 1.95 95%CI = 1.11–3.40	*p* = 0.011 * Exp(B) = 2.10 95%CI = 1.19–3.72 N = 0.076 ^a^	*p* = 0.118	-	*p* = 0.233	-
TCZ	*p* = 0.003 * OR = 2.37 95%CI = 1.32–4.25	*p* = 0.058	*p* = 0.000 * OR = 3.79 95%CI = 1.72–8.37	*p* = 0.001 * Exp(B) = 3.75 95%CI = 1.67–8.41 N = 0.125 ^a^	*p* = 0.063	-
RDV/TCZ	*p* = 0.031 * OR = 2.06 95%CI = 1.05–4.04	*p* = 0.159	*p* = 0.004 * OR = 3.35 95%CI = 1.42–7.88	*p* = 0.007 * Exp(B) = 3.36 95%CI = 1.40–8.07 N = 0.105 ^a^	*p* = 0.150	-
RDV/ANK	*p* = 0.187	-	*p* = 0.045 * OR = 2.49 95%CI = 0.99–6.23	-	*p* = 0.105	-
FPV/ANK	*p* = 0.183	-	*p* = 0.855	-	*p* = 0.263	-
FVP/TCZ	*p* = 0.039 * OR = 2.21 95%CI = 1.02–4.76	*p* = 0.118	*p* = 0.004 * OR = 3.70 95%CI = 1.44–9.46	*p* = 0.010 * Exp(B) = 3.51 95%CI = 1.34–9.21 N = 0.100 ^a^	*p* = 0.019 * OR = 3.86 95%CI = 1.16–12.89	*p* = 0.028 * Exp(B) = 3.86 95%CI = 1.16–12.89 N = 0.037
3MED	*p* = 0.001 * OR = 2.44 95%CI = 1.42–4.19	*p* = 0.001 * Exp(B) = 2.45 95%CI = 1.41–4.25 N = 0.094 ^a^	*p* = 0.001 * OR = 4.26 95%CI = 1.76–10.3	*p* = 0.001 * Exp(B) = 4.36 95%CI = 1.78–10.69 N = 0.140 ^a^	*p* = 0.054	-

* Statistically significant (*p* < 0.05); ^a^ The logistic regression model included age (negative coefficient); 95%CI, 95% confidence interval; Exp(B), exposure coefficient; N, Nagelkerke coefficient; OR, odds ratio.

## Data Availability

The data presented in this study are available on request from the corresponding author.
